# Reactive oxygen FIB spin milling enables correlative workflow for 3D super-resolution light microscopy and serial FIB/SEM of cultured cells

**DOI:** 10.1038/s41598-021-92608-y

**Published:** 2021-06-23

**Authors:** Jing Wang, Steven Randolph, Qian Wu, Aurélien Botman, Jenna Schardt, Cedric Bouchet-Marquis, Xiaolin Nan, Chad Rue, Marcus Straw

**Affiliations:** 1 Thermo Fisher Scientific, 5350 NE Dawson Creek Drive, Hillsboro, OR 97124 USA; 2 Knight Cancer Early Detection Advanced Research Center, Oregon Health and Science University, 2720 S. Moody Ave., Portland, OR 97201 USA; 3 Center for Spatial Systems Biomedicine, Oregon Health and Science University, 2730 S. Moody Ave., Portland, OR 97201 USA; 4 Department of Biomedical Engineering, Oregon Health and Science University, 3303 S. Bond Ave., Portland, OR 97239 USA; 5 Present Address: Allen Institute, 615 Westlake Ave N, Seattle, WA 98109 USA; 6 Present Address: Applied Physics Technologies, 1600 NE Miller St, McMinnville, OR 97128 USA

**Keywords:** 3-D reconstruction, Scanning probe microscopy, Super-resolution microscopy

## Abstract

Correlative light and electron microscopy (CLEM) is a powerful tool for defining the ultrastructural context of molecularly-labeled biological specimens, particularly when superresolution fluorescence microscopy (SRM) is used for CLEM. Current CLEM, however, is limited by the stark differences in sample preparation requirements between the two modalities. For CLEM using SRM, the small region of interest (ROI) of either or both modalities also leads to low success rate and imaging throughput. To overcome these limitations, here we present a CLEM workflow based on a novel focused ion beam/scanning electron microscope (FIB/SEM) compatible with common SRM for imaging biological specimen with ultrahigh 3D resolution and improved imaging throughput. By using a reactive oxygen source in a plasma FIB (PFIB) and a rotating sample stage, the novel FIB/SEM was able to achieve several hundreds of micrometer large area 3D analysis of resin embedded cells through a process named oxygen serial spin mill (OSSM). Compared with current FIB mechanisms, OSSM offers gentle erosion, highly consistent slice thickness, reduced charging during SEM imaging, and improved SEM contrast without increasing the dose of post-staining and fixation. These characteristics of OSSM-SEM allowed us to pair it with interferometric photoactivated localization microscopy (iPALM), a recent SRM technique that affords 10–20 nm isotropic spatial resolution on hydrated samples, for 3D CLEM imaging. We demonstrate a CLEM workflow generalizable to using other SRM strategies using mitochondria in human osteosarcoma (U2OS) cells as a model system, where immunostained TOM20, a marker for the mitochondrial outer membrane, was used for iPALM. Owing to the large scan area of OSSM-SEM, it is now possible to select as many FOVs as needed for iPALM and conveniently re-locate them in EM, this improving the imaging throughput. The significantly reduced dose of post-fixation also helped to better preserve the sample ultrastructures as evidenced by the excellent 3D registration between OSSM-SEM and iPALM images and by the accurate localization of TOM20 (by iPALM) to the peripheries of mitochondria (by OSSM-SEM). These advantages make OSSM-SEM an ideal modality for CLEM applications. As OSSM-SEM is still in development, we also discuss some of the remaining issues and the implications to biological imaging with SEM alone or with CLEM.

## Introduction

The advent of fluorescence microscopy has revolutionized biological sciences due to the chemical specificity with which structures may be targeted and visualized^1,2^. However, the resolution of conventional fluorescence microscopy is typically 200–500 nm, which is insufficient to resolve many components of biological ultrastructure typically sub-nanometer in size. Super-resolution microscopy (SRM) was awarded the 2014 Nobel Prize in Chemistry for improving the resolution of fluorescence imaging to tens of nanometers^3–7^. However, the resolution with which fluorescent biomolecules is imaged has limited value in the absence of the ultracellular context in the immediate proximity to the fluorescent markers. Scientists have frequently begun turning to electron microscopy to fill this contextual gap in a process known as correlative light and electron microscopy (CLEM)^8–10^. CLEM—the integration of two microscopic imaging modalities (light and electron microscopies) performed on the same sample—produces results that emphasize the strengths of each technique while offsetting their individual weaknesses.

In the past decade adoption of CLEM in biological studies has begun to accelerate due to development of SRM techniques, with improved fluorescent probes, sample preparation methods, and data processing software. These advances now routinely allow for imaging of proteins and organelles with the ultracellular information that is missing from fluorescence alone and with molecular specificity not easily achieved by electron microscopy (EM) alone^11–13^. Extending these techniques into three-dimensions offers the potential for revolutionizing data richness from CLEM imaging^10,14^. The cost of this additional information is usually an increase in the complexity of both sample preparation and the imaging technique. Until the development of iPALM, the resolution anisotropy of 3D SRM imaging techniques also limited many 3D CLEM experiments. In addition, acquisition of large volume, high resolution serial SEM datasets can become time prohibitive and may be compromised due to charging of the resin block face.

High-content 3D EM imaging techniques such as serial section TEM^15,16^, serial block face scanning electron microscopy (SBF/SEM)^17,18^ and focused ion beam scanning electron microscopy (FIB/SEM)^19,20^ have been applied to a diverse array of biological applications. Recently, gas cluster ion sources have been utilized in serial ion erosion/SEM imaging in a spinning sample configuration to generate 3D reconstructions of large tissue volumes^21^. This work is noteworthy in that it somewhat bridges a gap between FIB/SEM and SBF/SEM where the precision of ion milling is combined with the large volumes afforded by SBF/SEM. It is also a departure from standard ion erosion techniques in that it leverages a low energy cluster ion source, which improves cut face quality over Ga^+^ FIB.

The recent commercial availability of ThermoFisher Scientific’s Hydra™ (multiple ion species PFIB) offering different primary ion species (Xe^+^, Ar^+^, O^+^ and N^+^) has opened a completely new space to utilize FIB/SEM for applications that do not respond well to conventional Ga^+^ FIB or even SBF/SEM. Given there are decades of experience behind reactive ion etching with oxygen plasma, we presumed that a focused oxygen ion beam may offer many of the favorable, chemically enhanced substrate erosion characteristics of its broad area predecessor. This was first successfully used to directly fabricate and evaluate damage layers in optically active diamond structures using chemically-enhanced oxygen FIB^22^ as opposed to Ga^+^, which quenches the optical properties. More recently oxygen FIB has been highlighted for use in life science research directed at resin-embedded cells and tissue^23^. Of note in this work was a comparison of various focused ion species applied to an array of resin types. Here, oxygen provided clear improvement in cut face quality and image contrast was evident, as well as an overall agnosticism to resin type.

Here we present a novel three-dimensional CLEM workflow in flat-embedded, cultured cells, which utilizes this focused oxygen ion beam as the eroding medium in a recently developed reactive FIB/SEM process. For the SRM imaging portion of the workflow, we employ iPALM^24,25^ , which has improved the resolution of fluorescence light microscopy to ~ 10 nm in both axial and lateral dimensions. This nearly isotropic spatial resolution approaches that of biological EM, making iPALM well suited for CLEM. Correlative FIB/SEM and iPALM has been demonstrated using more conventional Ga^+^ FIB approach^10^ to address about 30 um × 30 um × 10 um targeted volume. We aimed to develop a workflow that would take advantage of iPALM 3D resolution, but also be able to address a larger ROI (hundreds of um in x–y sample plane) for statistical purposes with improved contrast and resolution in the SEM phase. Strict sample geometry and environmental conditions are placed on iPALM so the technique developed and presented here works within these constraints, but the workflow can readily be applied to other SRM modalities. For EM imaging, we incorporate a reactive oxygen focused ion beam in a PFIB DualBeam system to perform large area, glancing angle ion erosion of a rotating resin-embedded sample, a strategy we term OSSM. The result is a series of SEM images each representing a thickness ranging from 5 to 10 s of nm in the axial direction and as much as 1 mm diameter in the lateral direction (i.e. the plane of the cell culture surface). The large area and fine slice thickness (as controlled by oxygen ion dose) are very well matched to the single layer cell cultures that are used in iPALM and many other SRM techniques. The combination of the two modalities thus allows 3D imaging with molecular specificity, nanometer resolution, as well as the capacity to address hundreds of cells in a single experiment. Additional benefits of reactive oxygen over inert ion sources are reduced sample charging and enhanced image contrast, both of which allow minimal staining to be employed. This also lowers the risk of significantly altering the ultrastructure of the cell between the iPALM/SRM imaging and OSSM steps, a unique advantage over other FIB/SEM and SBF/SEM techniques. Lastly, while the workflow is tailored for correlation with iPALM, its extension to other SRM and conventional light microscopy modalities should be straightforward.

## Results and discussion

**Figure 1:**
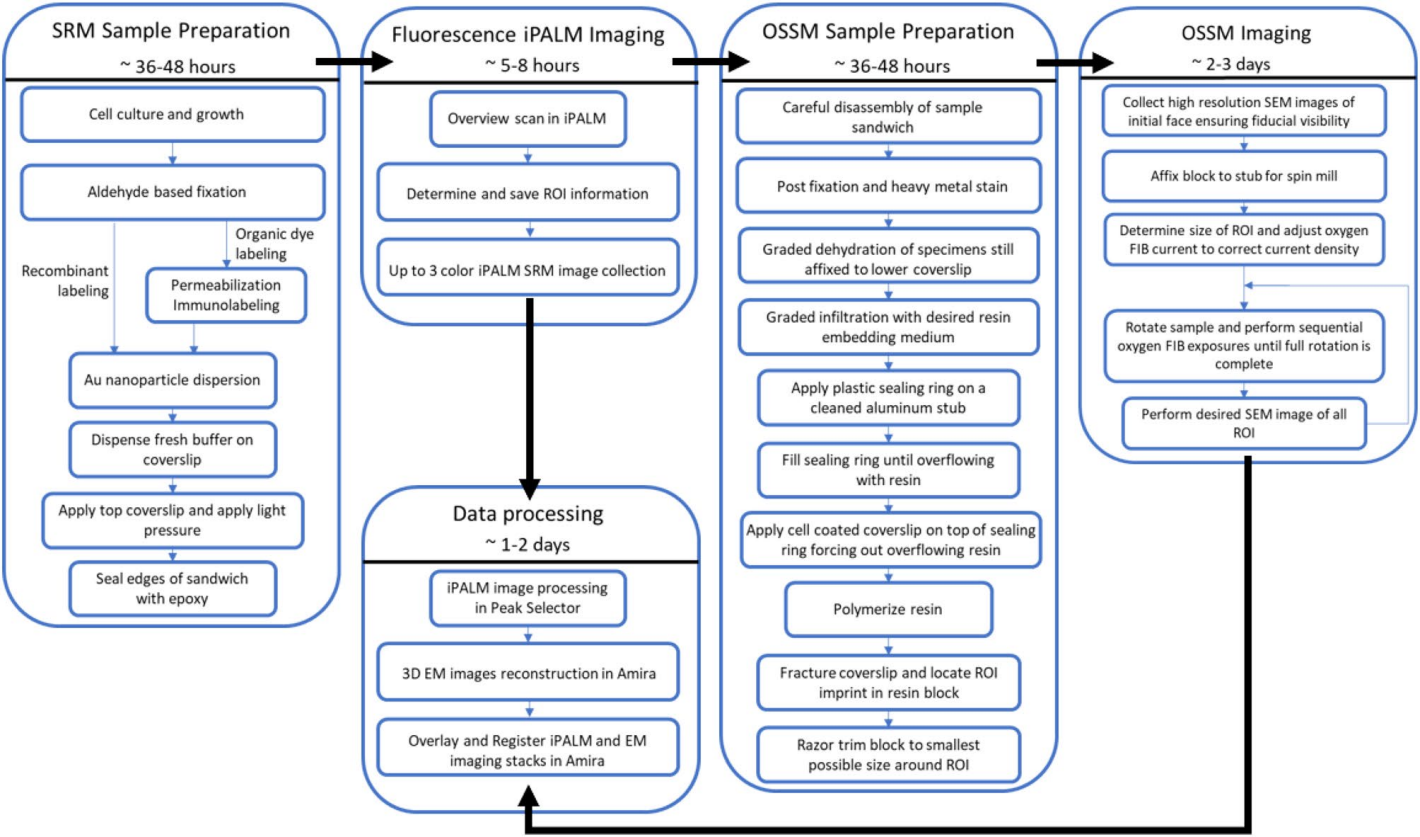
Quick-reference flowchart describing the correlative iPALM / OSSM workflow with time estimates for each phase of the workflow. Some data processing may be done in parallel to reduce the overall time to data by approximately one day.

The example workflow outlined in this paper for high-resolution 3D CLEM involve the use of a single SRM imaging modality paired and correlated with our OSSM spin milling serial SEM imaging technique. Intended to be a quick reference, Fig. 1 is a concise flowchart of key steps in the entire workflow beginning with cell culture and ending with post-processing of the data. To aid in planning, time estimates of each phase of the workflow are given. In the subsequent sections, we present in detail the methods outlined in Fig. 1.

In development of a workflow, it is necessary to demonstrate on a well understood sample to confirm the results are as expected from existing literature. For this we chose to immunolabel TOMM20, a transport protein located on the mitochondrial outer membrane, in cultured human osteosarcoma cells (U2OS). Cells were plated on photoetched glass coverslips for 48–72 h, followed by fixation and immunolabeling as described in “SRM (iPALM) sample preparation” section. Next, we acquired 3D SRM fluorescence data on a custom iPALM setup, which was the first iPALM commercial prototype built at ThermoFisher Scientific, as described in “Fluorescence iPALM imaging” section ‘Imaging Protocols’. Following iPALM imaging, the cells were flat embedded in resin (“OSSM sample preparation” section) to prepare for the OSSM process. We then performed OSSM over an area encompassing all the iPALM-imaged regions to generate a series of 2D SEM images as outlined in “OSSM for serial PFIB/SEM imaging” section. Finally, 3D iPALM images and the corresponding SEM volumes were reconstructed and registered (“Data processing” section). As such, we give a successful demonstration of a full 3D CLEM workflow by showing the correct localization of TOMM20 (determined with iPALM) precisely to the outer membrane of mitochondria (resolved with OSSM) in the rendered volumetric images with minimal structural distortions during sample preparation and imaging.

### SRM data

**Figure 2:**
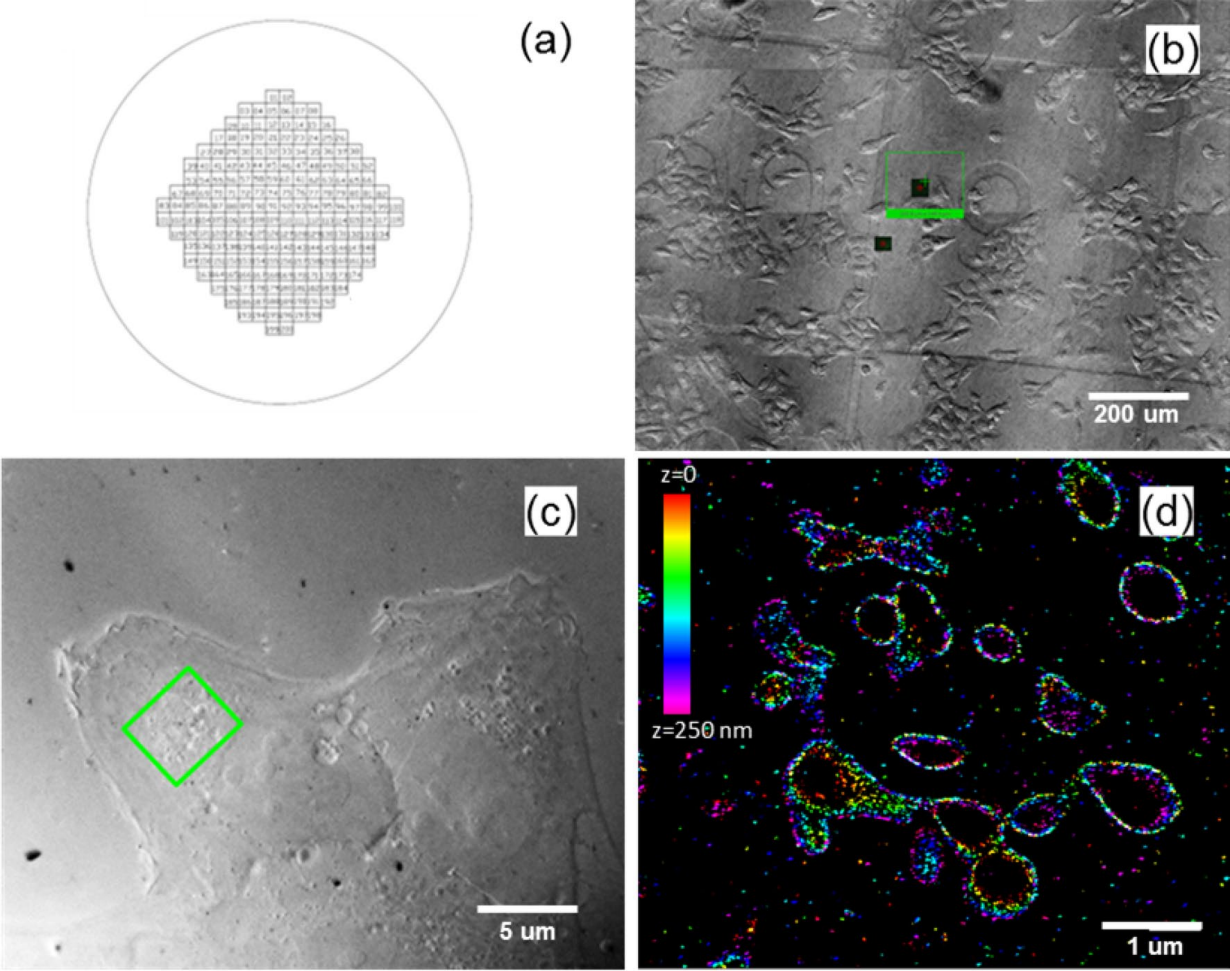
Schematic of 25 mm diameter photoetched coverslip (**a**); Brightfield image of the sample under prescan mode (low magnification) of iPALM system (**b**). Brightfield image of the target ROI under iPALM mode (high magnification) of iPALM system (**c**). color coded 3D iPALM fluorescence imaging within 250 nm z depth (**d**).

SRM based on the iPALM technique requires fluorophores that can undergo stochastic switching between a fluorescent and a non-fluorescent state. By making use of photoswitchable fluorophores previously validated for single-molecule SRM, iPALM employs similar labelling strategies, such as immunolabeling and fluorescent protein tagging. A key advantage of immunolabelling for fluorescence imaging is that it allows both the detection of endogenous proteins and the use of bright organic fluorophores with efficient switching, which leads to higher single molecule localization precision. U2OS cells cultured on photoetched glass coverslips (Fig. 2a) were fixed and immunolabeled, after which imaging buffer was applied and the sample sandwich sealed as described in “SRM (iPALM) sample preparation” section. Figure 2 contains optical micrographs of multiple cells (Fig. 2b) and a single targeted cell (Fig. 2c) for iPALM analysis. The first important frame of reference to make note of is the numbered grid into the coverslips, shown schematically in Fig. 2a and also seen in the optical micrograph of Fig. 2b. Since this pattern is photoetched, it served as a template for transfer into resin and was used to re-locate the ROI in the OSSM process. Gold nanorods (57 × 25 nm) immobilized on the coverslip were used as markers for both iPALM alignment (e.g. between the top and bottom objectives) and z-calibration in the interferometric process, both critical to the optimal iPALM performance. Z-calibration was carried out for each ROI prior to actual image acquisition, by obtaining interferometric images of the gold fiduciaries on the three cameras while scanning the axial position of the sample with a piezoelectric stage. Next, the cells of interest were imaged by collecting tens of thousands of raw, single-molecule images while the fluorophores underwent dynamic photoswitching. The resulting raw image stacks were then processed to obtain the precise molecular localizations, based on which a 3D image of the target was then reconstructed (Fig. 2d). Details of the iPALM imaging process can be found in “Fluorescence iPALM imaging” section and elsewhere^24^. Given this is a single-color experiment, there is only a single molecular label so there is no contextual information at this point. There are effectively only outlines of mitochondria visible (Fig. 2d). Their spatial location on the sample can only be reference to the fluorescent gold nanorods and the etched grid visible in the optical micrograph.

### OSSM FIB/SEM data

The next phase of the correlative workflow is disassembly of the sample sandwich and preparation for OSSM FIB/SEM, which occurs over several days as outlined in “OSSM sample preparation” section. Although very recent in development, the use of oxygen PFIB for spin milling has several demonstrated advantages when applied on resin embedded cultured cells especially when coupled with SRM for CLEM. Based on the ability of reactive ions to form strong chemical interactions with the substrate, research began uncovering that focused, reactive ion sources—in particular, oxygen—could be useful on a range of carbonaceous substrates including polymers, resin^23^, diamond^22^, and biological tissue. The milling results in chemically enhanced sputter rates, smoother surface texture, reduced charging and less beam-induced damage^22,23^. When oxygen PFIB is applied to embedding resins and polymers the unique chemical interaction also offers a much broader sample compatibility compared to “standard” FIB/SEM and SBF/SEM. But top-down cross-sectioning of samples must remain very site-specific as the milling of millimeter-size cross-sections becomes time prohibitive. Also, top-down cross-sectioning—even with oxygen PFIB—increases the chance of curtaining artifacts, which may distort the target ROI. OSSM alleviates these problems by milling from many different incident angles over a large area and small z-depth.

**Figure 3:**
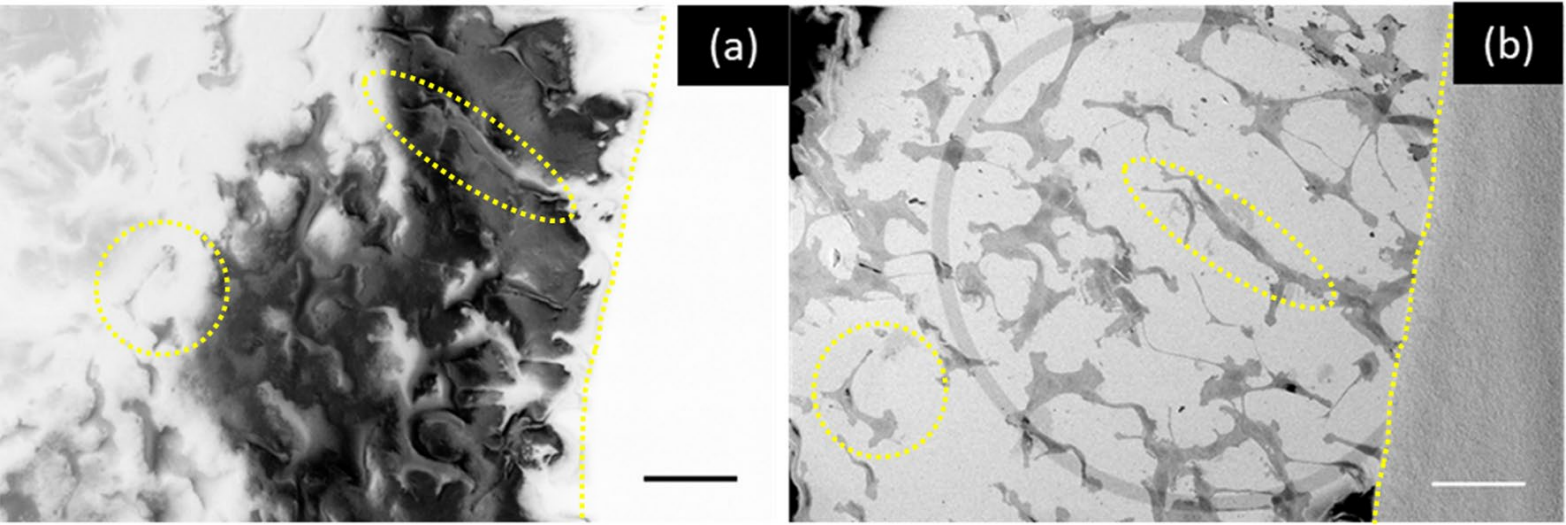
Charge dissipation and contrast enhancement on resin embedded U2OS cells after oxygen beam irradiation. Pre-irradiation (**a**) shows substantial charging and cellular contrast loss, which is greatly improved after irradiation (**b**). Scale bar : 100 µm. Circled regions indicate the same features on the sample without (**a**) and with (**b**) oxygen FIB exposure. The dotted line marks the border between carbon paste and sample.

Additionally, the reactive ion source fundamentally performs better on resin embedding media resulting in improved SEM image quality. Figure 3 contains a before and after image pair showing the charge dissipation and contrast enhancement when oxygen PFIB exposure during OSSM is applied to embedded U2OS cells. While we do not have a full understanding of the mechanisms involved at this point, the qualitative differences in the two images are clear. Due to the excessive charging of the resin sample, in order to even visualize any cells, the secondary electron detector gain must be maximized to the point that the carbon paste (right side of images) is completely washed out in Fig. 3a. For resin samples with severe charging, platinum or carbon conductive layer coating is often necessary to obtain useful images^19^. With OSSM, a 12 kv, 65 nA oxygen beam was applied on the 500 µm × 500 µm sample area with -35 degree stage tilt. Six PFIB patterns were performed at different stage rotations (60 degree increment) with 20 s of FIB milling time for each pattern. Charge dissipation was observed after 2-min oxygen PFIB exposure. After this exposure the detector gain may be reduced so that much more grayscale information is available in the images across the entire field of view. Additionally, sample charging commonly results in dynamic variations in the images, for examples beam drift and contrast reversals, which is not captured in the still images. These effects collectively contribute to the image enhancement after oxygen exposure.

The samples were embedded using Embed-812 resin due to its excellent stability under electron beam irradiation and the fact that we do not need to be concerned with fluorescence preservation. However, with OSSM, the sample preparation can be flexible, allowing for different resin types and fixation/contrast enhancement protocols, for example, when fluorescence preservation is desired. In a large part owing to the reduced charging, sample stability, and enhanced image contrast, we found that OSSM-SEM also performs well on samples with reduced doses of osmium post-fixation and uranyl acetate staining as compared to other EM sample preparations (data not shown). This could be a major benefit for those CLEM applications that require little to no osmium staining or postfixation, for example when using methacrylate or LR white resins^26^.

**Figure 4:**
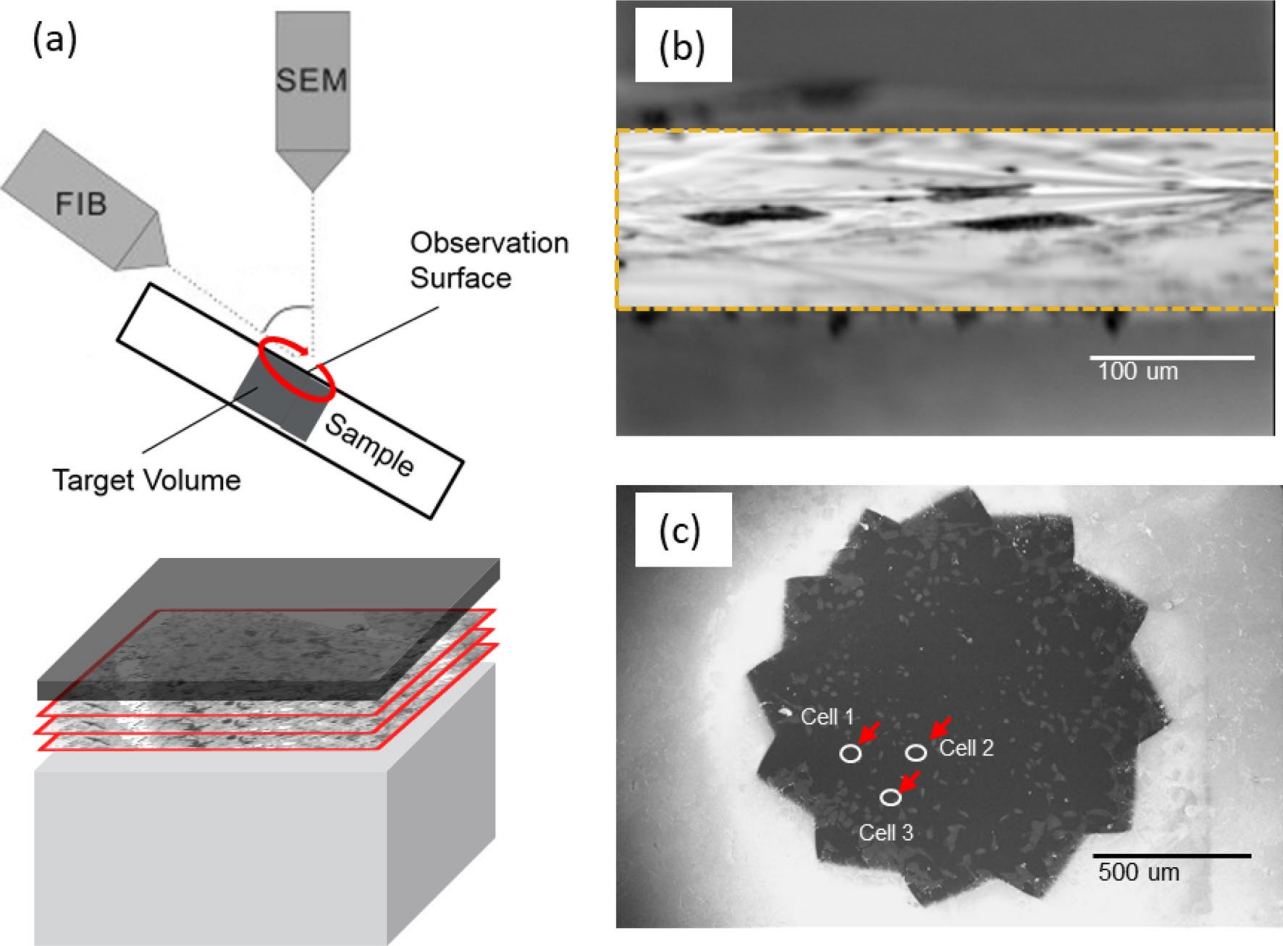
Schematics (**a**) showing key system and sample geometry associated with OSSM. Lower portion of (**a**) shows how a 3D volume is created with plan-view slicing in this geometry. Scanning ion micrograph (**b**) of the pattern area when performing a single iteration of the OSSM slice mill. SEM image (**c**) of the milled slice after 12 PFIB patterns at different stage rotation (30° increments) has been performed. This series of rotations and mills approximates a continuously rotating ion polish and constitutes one slice of the OSSM workflow. Red arrows indicate three ROIs that were imaged by iPALM.

For serial image collection using OSSM, the sample was mounted with the ROI roughly centered on the PFIB DualBeam’s rotational axis and tilted to nearly glancing angle as shown schematically in Fig. 4a. Figure 4b is a scanning ion micrograph showing the patterned area at high tilt. Figure 4c is a plan-view SEM image of a single OSSM slice performed on the block face.

**Figure 5:**
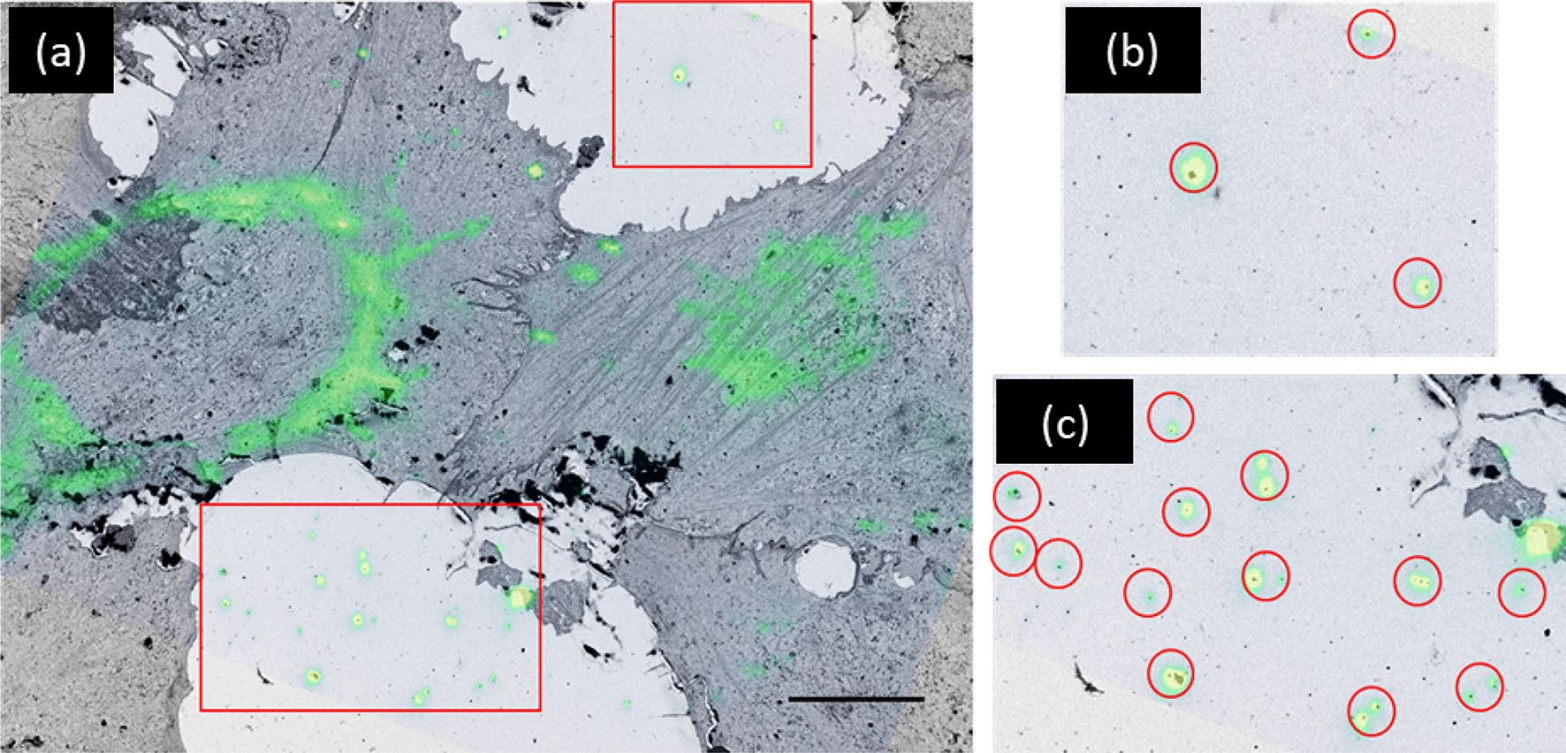
Overlaid iPALM 2D projection fluorescence image and SEM image used for coarse co-registration. Gold nanorods are visible in both imaging modalities as shown in (**a**). Higher magnification images of the two highlighted regions of fiducials are shown in (**b**) and (**c**). The broad area of OSSM ensures many fiducials may be used for correlation. Scale bar in (**a**) is 10 µm.

An added benefit of OSSM is that it maximizes the preservation of fiducial marks that are critical to co-registration and correlation of the data sets. All the fiducials (both gold nanorods and the grid imprint) are located in the sample plane at the surface of the block face. Working over large areas ensures that more fiducials can be used for co-registration of the iPALM and SEM data. Figure 5 shows an example set of SEM and 2D projection iPALM images overlaid showing the ability to correlate locations of many fiducials simultaneously that are in the same sample plane in both imaging modalities. An abundance of fiducials is extremely useful in the case that some do not fluoresce or are lost during sample preparation. The large area scan makes it possible to acquire as many ROIs as needed during the SRM (iPALM) imaging stage, all of which may be covered within a single, subsequent OSSM-SEM session. This is not yet possible with current CLEM workflows based on conventional FIB/SEM—SRM. Although we demonstrated only three iPALM ROIs in this paper due to limited SRM buffer lifetime, addressing such a large area inherently allows hundreds of cells to by analyzed simultaneously in a single experiment.

**Figure 6:**
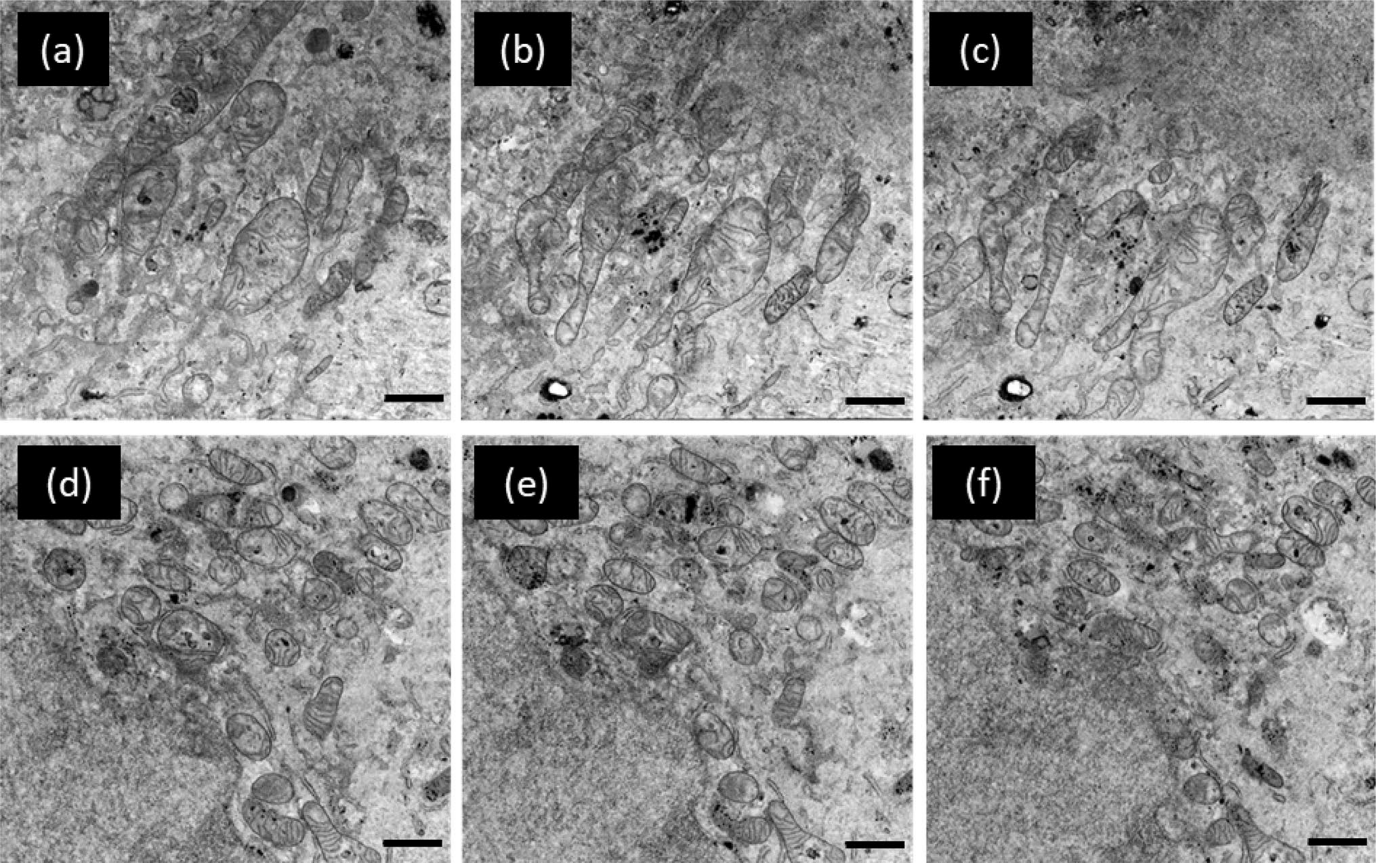
Select SEM images of slices of nominal 5 nm thickness taken from the OSSM data set on targeted cells. Images (**a**–**c**) are one subset of cells at slice 35, 55, 75. Images (**d**–**f**) are a second subset of cells imaged on the same slice as (**a**–**c**) Scale bar: 1 µm.

After collection of the initial block face images to locate ROIs and identify gold fiducial markers, the serial process of stage rotation, oxygen PFIB exposure, and SEM imaging was automated and allowed to complete. Figure 6 contains SEM images of two regions of interest at three different slice increments (slice 35, 55, and 75) taken during the OSSM process. These are later used to reconstruct the EM data into renderings of 3D volumes. In this experiment, a total of 61 slices were performed with a nominal thickness of 5 nm each within 25 h. High-res SEM images were acquired from three different ROIs with 40 um horizontal field view to correlate with fluorescence data.

Embed-812 resin prepared as described in “OSSM sample preparation” section did not show any major deformation or melting defects during FIB milling. Small differences that can be observed as we overlay the iPALM and EM images are most likely due to the deformation of the sample occurring during the dehydration and embedding step as it is very commonly reported to occur in EM preparation. Care should be taken in sample preparation to minimize these effects if possible. Another source of error that should be noted is that the shrinking of the sample is usually anisotropic and can be difficult to correct during the experiment and in data reconstruction. In our work, this type of anisotropic deformation was not obvious for the structures we were analyzing.

Through experimentation, we discovered several advantages to applying OSSM to our CLEM workflow. Firstly, it is not necessary to perform trimming and microtome sectioning on the sample after resin block polymerization, as milling and imaging begin at the block surface closest to the etched patterns, cells, and fiducials. Because the very first interface at the block face is preserved and the first slice removed may be only a few nanometers, it is readily feasible to keep the ultrastructure and small features proximal to the bottom plasma membrane both intact and continuous. Secondly, the usual preparation steps associated with conventional FIB cross-sectioning and SEM imaging (excavating large volumes around the target volume, depositing protective caps and depositing fiducials) can be dispensed, while still being able to achieve smooth surface textures on targeted ROI with minimal curtaining artifacts. Thirdly, the milling slice thickness can be kept highly consistent by controlling the ion dose, which is easier than controlling the spot size or beam profile in standard FIB/SEM, top-down cross-sectioning. We achieve 5 nm to tens of nm slice thicknesses in our experiments, and simultaneously keep a large horizontal field of view. Lastly, the slice orientation of the OSSM technique was the same as iPALM imaging data. This results in a simpler ROI relocation step.

It is important to note that other well-established EM modalities used in CLEM, for example Ga^+^ serial FIB/SEM, serial block face imaging on VolumeScope^27^, are also compatible with our workflow. However, OSSM is particularly well matched to iPALM imaging constraints and resolution. For instance, VolumeScope SBF imaging is a robust way to acquire large volume in reasonable time, but additional sample preparation and staining is needed. Moreover, z-resolution is limited due to the cut resolution of the ultramicrotome diamond knife. Ga^+^ FIB-SEM imaging is very site specific and can reach a few nanometers of axial resolution. However, the imaging volume size (usually tens of micrometers in all three dimension) is extremely limited, and imaging time may be cumbersome depending on the imaging area. In the end, the researcher will have to evaluate the best instrument for their cells/tissues under investigation and the features of interest to be imaged, but the flexibility of this sample preparation workflow to extend to many other modalities lends itself to a wide array of specimens and applications.

### 3D reconstruction and correlation

**Figure 7:**
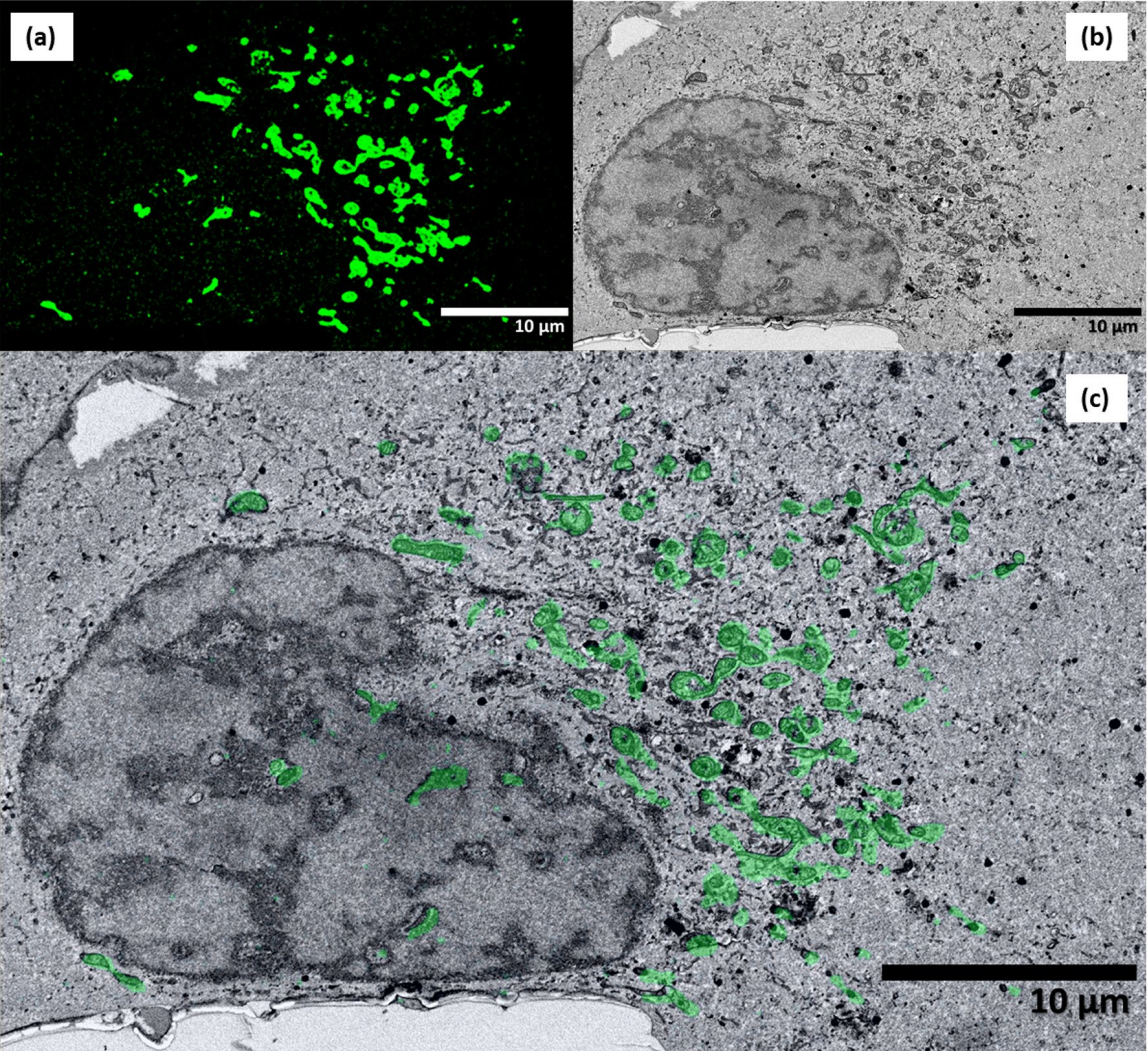
Two-dimensional projection of iPALM-imaged single U2OS cell with outer mitochondrial membrane immunolabeled with TOMM20 (**a**). Single SEM of a single slice of the OSSM process where ultrastructural detail in the cell is evident (**b**). Overlay of the 2D iPALM projection onto the single OSSM slice showing a correlation between mitochondrial structures and the TOMM20 label (**c**).

**Figure 8:**
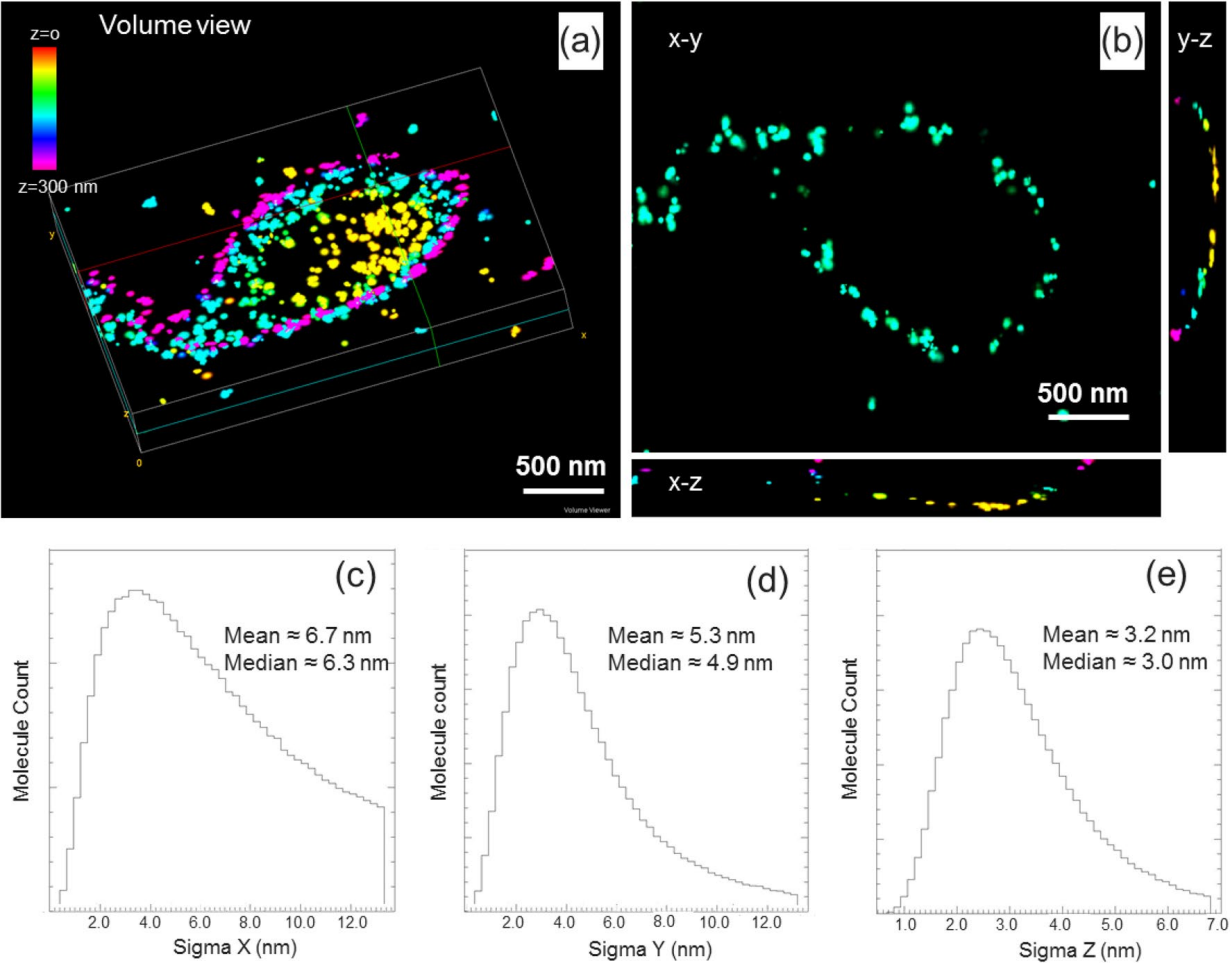
(**a**,**b**) 3D renderings of the outer mitochondrial membrane of the targeted U2OS cells. Several vantage points are shown to illustrate the nearly continuous nature of the outer membrane. Single molecule localization accuracies in x, y, z were shown in (**c**–**e**) as calculated by Peakselector. The cutoffs for Sigma X, Sigma Y, and Sigma Z were ~ 13 nm, ~ 13 nm, and ~ 7 nm, respectively. The pixel size of the imaging system is 133 nm/pixel.

After iPALM and OSSM data collection, the process of image reconstruction is the next step in creating a correlative 3D volume. PeakSelector, a single molecule localization software package developed by Shtengel et al*.* (described elsewhere^24^) is used to extract the fluorescence events and assign them to molecules. Since the z-axis information has not been computed at this point, the image represents a 2D projection of all the molecules identified by the iPALM localization software. The iPALM localizations shown in the overlay of Fig. 5a (green overlay) and in Fig. 7a are such 2D projections. Figure 7a is representative of a single cell region where only the outer membranes of mitochondrial structures are visible. Figure 7b,c show the SEM and overlay of the 2D iPALM on a single OSSM slice, where the 2D projection is useful for a rough evaluation of the alignment between the iPALM and the SEM images. However, more information is present in the iPALM data: the signal from each molecule forms a different interference pattern, recorded as a unique combination of intensities on the three cameras, depending on the molecule’s location in z-space. These interference patterns are compared to the z-calibration data taken on the gold fiducials and from this the axial position of each molecule is extracted. Additional information on the theory and practical aspects of iPALM can be found in Shtengel et al.^24^ Using this z-extraction procedure, 3D images of the outer mitochondrial membranes—as highlighted by immunolabeled TOMM20—were generated and are shown in various viewing perspectives in Fig. 8. Here the nearly continuous nature of the outer membrane is evident, and PeakSelector indicates that x, y, z localization accuracies are all better than 10 nm. The high precisions are due to the double photon yield (x, y) and single-photon interferometry (z), both benefits of using a dual-objective setup.

**Figure 9:**
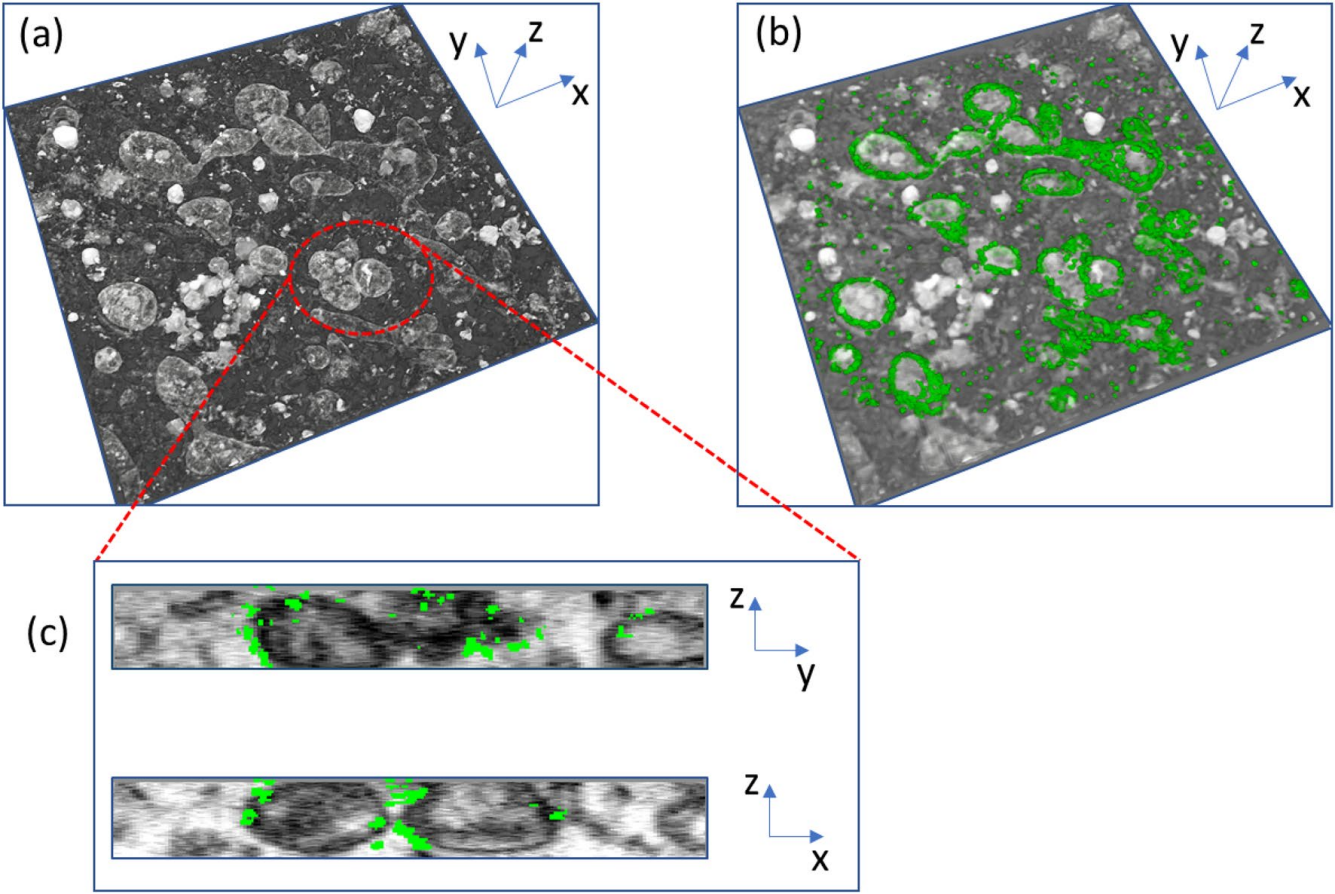
Full 3D CLEM dataset showing 3D volume renderings of OSSM SEM data (**a**) and with 3D iPALM rendering overlaid (**b**). OSSM SEM reconstruction and overlaid iPALM data of targeted mitochondria in YZ and XZ space (**c**). TOMM20 fluorescent markers are found only at the outer membrane of the mitochondrial structures as expected.

OSSM SEM data contains a series of 2D images (like shown in Fig. 7b) that must be aligned and rendered into a continuous volume based on the a priori knowledge of the nominal slice thickness. High resolution SEM images of each ROI are collected and used as reference images for subsequent slice alignment. This was most easily carried out using the commercially available Amira software package. The images were imported, aligned, digitally processed, and used to render 3D volumes. Manual registration based on fiducial identification in each image modality was used to coarsely overlay the two datasets. As a fine adjustment more manual registration was carried out using the large amount of contextual information available in the OSSM SEM images. The culmination of the workflow produced a 3D CLEM dataset shown in Fig. 9 where there are clear correlations between the 3D iPALM fluorescence data and the well-known EM depiction of mitochondrial membrane structure.

## Materials and methods

In this paper, we utilize the iPALM technique as our preferred 3D SRM modality and present methods to prepare samples for this initial imaging step while allowing correlation with OSSM SEM image data sets of resin embedded cells.

In iPALM, the axial localization of molecules relies on interferometry, which is highly sensitive to the differences between the light paths. This enables z-resolution of approximately 10 nm, which is among the best of all existing SRM techniques to date. The x, y-resolution is also typically 10–20 nm. This isotropic, high spatial resolution is similar to that achieved with biological SEM, making iPALM well suited for correlative imaging with 3D SEM.

That being said, current iPALM setups and procedures are much more complicated than other single-molecule SRM modalities, which places constraints on how samples are prepared and, correspondingly, how the correlative process must be carried out. In general, it is required that the regions of interest be re-located and re-imaged with EM after they have been imaged in iPALM first. In between iPALM and EM is a lengthy sequence of sample preparation steps including post-fixation, resin-embedding, and heavy metal staining. For obvious reasons, all the EM sample preparation steps that occur after iPALM imaging need to be done in a way that best preserves the sample structure. Another challenge faced by iPALM-CLEM is the development of sample preparation protocols that allow optimal performance of both imaging modalities. Strong fluorescence signal and low background are necessary for high quality iPALM imaging performance. Additionally, good photo-switching properties are required of the fluorescent probe to achieve single molecule localization^28^. For an optimal CLEM workflow, EM sample preparation protocols, including strong chemical fixation, dehydration/infiltration, and heavy metal staining, need to be considered carefully as to not degrade the SRM imaging conditions. In addition, alignment and co-registration of the two data modalities (iPALM and EM) with high accuracy in 3D space is vital.

To this end, we have successfully created a correlative workflow in which iPALM imaging of immunolabeled U2OS cells is performed prior to the resin embedding process. The advantage to performing iPALM in the pre-embed phase is that it allows optimization of the fluorescence SRM imaging conditions with minimal consideration to the EM sample preparation that follows. We performed minimal membrane permeabilization during the sample immunolabeling to avoid cellular structure extraction as preferred in most EM sample preparation protocols.

A major challenge in the latter steps of the workflow is the requirement of locating and aligning the same region of interest (ROI) between iPALM and SEM images. To overcome this issue, we first grow cells on photoetched round glass coverslips which have 200 alphanumerically labelled square regions arranged in a diamond pattern (Bellco Glass Inc. Part #: 1916–91,025) (Fig. 2a). The unique feature of these coverslips which makes them truly useful to this workflow is that the above described pattern is etched into the coverslips, making it possible for features of the pattern to be transferred to the resin block during subsequent flat embedding. Next, our iPALM microscope is equipped with an overview mode, which can generate a stitched image with low-magnification (20x) bright field imaging conditions (Fig. 2b). A zoom-in ROI can then be selected for further iPALM imaging at higher magnification. Once the ROI has been specified, the sample is shuttled from overview mode to iPALM mode via an automated translation stage. A bright field image can be taken of this ROI with the 60 × objective to further probe interesting cell morphologies (Fig. 2c).

### SRM (iPALM) sample preparation

**Figure 10:**
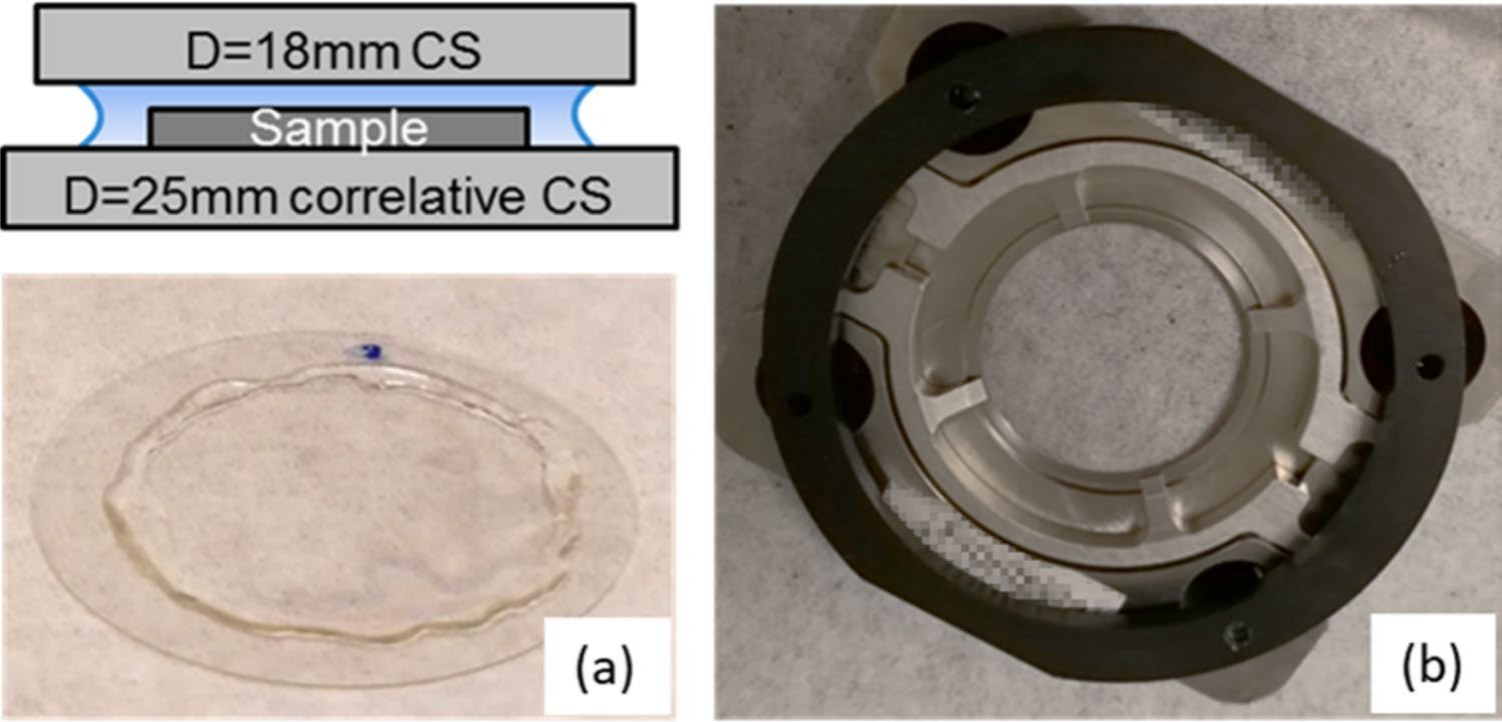
The “sample sandwich” schematic (upper) and photograph (lower) configuration where cultured cells are grown on the lower coverslip, then topped with buffer solution and a second coverslip (**a**). The sandwich is sealed with epoxy.; Sample holder for the iPALM system used to secure the sample sandwich and transfer it into the iPALM imaging path (**b**).

In the present work, we cultured U2OS cells (human osteosarcoma) at 37 °C and under 5% CO_2_ in Dulbecco’s Modified Eagle’s Medium (DMEM) supplemented with 10% fetal bovine serum (Life Technologies, 11,995 and 10,082, respectively). The cells were provided by Dr. Nan lab through a collaboration. The photoetched coverslips (BELLCO Glass Inc. Custom item) with #1.5 (~ 0.17 mm) thickness and 25 mm diameter were first sonicated in 100% Ethanol for 5 min, washed with phosphate buffered saline (PBS) (3 × 10 min), and left to dry and sterilize in a tissue culture hood with UV illumination for 30 min. Cells were plated on the coverslips which fit neatly into 6-well microplates and incubated for 24–48 h to reach about 70% confluency for imaging. Subsequently, cultured cells were fixed with a mixture of 3.7% paraformaldehyde (PFA) and 0.1% glutaraldehyde (GA) for 20 min followed by washing with 1 × PBS (3 × 5 min). Cells then were permeabilized and blocked in a mixture of 3% bovine serum albumin (BSA) and 0.1% Triton X-100 for 30 min. A primary antibody for TOMM20 (ab78547, Abcam, 1:500 dilution in PBS buffer with 3% BSA) was then applied to cells and incubated for 45 min at room temperature. Following removal of the primary antibody, the cells were washed in 1 × PBS (3 × 5 min). A AlexaFluor 647 conjugated anti-rabbit secondary antibody was incubated with the cells at a final concentration of 0.1 µM for 30 min while protected from light. The AlexaFluor 647 organic fluorophore exhibits complete photoswitching in the presence of a specialized imaging buffer, which was prepared by mixing Tris Normal (TN) buffer, glucose oxidase-containing buffer (GLOX), and β-mercaptoethylamine (MEA) solution. The TN buffer was made by dissolving 10% glucose (w/v, Fisher Chemicals D16-500) in 50 mM Tris buffer with 10 mM NaCl (pH 8.0). The GLOX buffer stock solution contained 0.5 mg/mL glucose oxidase (Sigma-Aldrich, G2133-50 kU) and 40 µg/mL catalase (Sigma-Aldrich, C100-50MG). MEA (Sigma-Aldrich, 30070) was dissolved at 1 M in 0.25 mM HCl. Prior to each experiment, the imaging buffer was freshly prepared by mixing GLOX, MEA, and TN at a ratio of 1:1:98 per reported protocols^29^. Fiducials for iPALM imaging–57 nm-length, 25 nm-diameter gold nanorods (A12-25–600, Nanopartz)—were suspended in Dulbecco’s PBS (DPBS) with Ca^2+^/Mg^2+^ and applied to the stained cells for 20 min, and then followed with one PBS wash prior to imaging. These fiducials were used for both sample spatial drift correction and as a source of the critical axial position (z-depth) interferometric calibration for iPALM. Lastly, to adapt the sample to the requisites for the iPALM setup, 10–15 ul of the imaging buffer was applied on the cells, then covered with a smaller (18 mm diameter) upper coverslip. Moderate thumb pressure is then applied to the upper coverslips until a relatively uniform interference pattern known as the Newton’s rings can be directly observed. Typically, when the number of rings observed is few, and the rings themselves broad, we assume that a uniform gap of approximately 10 µm between the upper and lower coverslips has been achieved and that the sample is ready to be sealed. This so-called “sample sandwich” is then sealed with 5-min epoxy applied around the rim of the upper coverslip (Fig. 10), effectively sealing in the buffer, preventing leakage, and limiting oxygen intrusion which can negatively impact the photo-activation properties of the fluorophore.

### Fluorescence iPALM imaging

iPALM data collection began with locating a cell of interest that has enough fiducials nearby to produce a reliable 3D calibration. Data generated during calibration on the gold fiducials determine how the interference images (recorded on three cameras) change with the axial position of each particle; the resulting ‘calibration curve’ is then used to determine the axial position of actual fluorophores in the subsequent image collection and reconstruction. After the sample was loaded and the ROI identified, several microscope adjustments are required. The first of these is the adjustment of the total internal reflection angle correction applied to the excitation beam. Since each sample is different, this is manually adjusted until maximum signal to noise is achieved in the fluorescence image. Next, the microscope must be optimized for each ROI and a z-calibration performed near the feature of interest. The process has been described earlier and may be found elsewhere^24^ in greater detail. Prior to iPALM image collection, the laser parameters and camera timings are set to desired values. For iPALM imaging of AF647, a 638 nm excitation laser (1–2 kW/cm^2^) and a 405 nm photoactivation laser (1–10 W/cm^2^) were used. The 405 nm laser was pulsed at 1/6 duty cycle to ensure appropriate switching rates of the AF647 fluorophores. At these laser power densities, images were acquired with 15 ms exposure time. Beyond this step, the data collection is automated with frames from all 3 cameras being stored to hard disk for the 50,000 frames.

### OSSM sample preparation

Following SRM image collection, the sample was removed from the iPALM sample holder. A syringe needle and razor blade were used to scrape the epoxy sealant away from the edge of the top coverslip. Care must be taken to not break the coverslip, and to avoid disruption and pressure on the imaged region. The top coverslip was then carefully removed, and the buffer was washed away with a series of dips into deionized water with mild agitation. Once the buffer was removed, the sample was placed in cold Karnovsky’s EM fixative (2.0% PFA, 2.5% GA in 0.1 M sodium cacodylate buffer, pH 7.2). After this fixation step, the samples were rinsed using 0.1 M sodium cacodylate buffer (3 × 5 min), then post-fixed in 2% reduced osmium tetroxide prepared in 0.1 M sodium cacodylate buffer and 1.5% K_3_Fe(Cn)_6_ for 30 min at room temperature. Cells were washed with Milli-Q water (3 × 5 min) and followed a 30-min incubation in 1% tetracaine hydrochloride (TCH) solution. To improve EM contrast, a second 30-min post-fixation of 2% OsO_4_ in deionized water was applied to the sample after washing away the TCH solution. Subsequently, cells were washed with Milli-Q water (3 × 5 min) and *en bloc* stained using saturated 5% uranyl acetate solution in water for 30 min. Once stained, the sample was subjected to a series of graded acetone dehydrations steps (30%, 50%, 75%, 90%, and twice 100% for 2 min each) and ultimately, infiltration with Embed 812 resin (Embed-812 36.75 g, DDSA 22.75 g, NMA 19.25 g, BDMA 2.1 mL) was initiated. Cells were infiltrated with a 1:1 mix of acetone and resin for 1 h and then 100% resin with three exchanges followed by an overnight incubation at 100% resin. All dehydration/infiltration was performed in a disposable dish which must be resistant to acetone and resin. Since fluorescence imaging has already been performed, we can increase the infiltration time in order to ensure adequate infiltration of the resin into the cells, thus improving the EM imaging conditions.

**Figure. 11:**
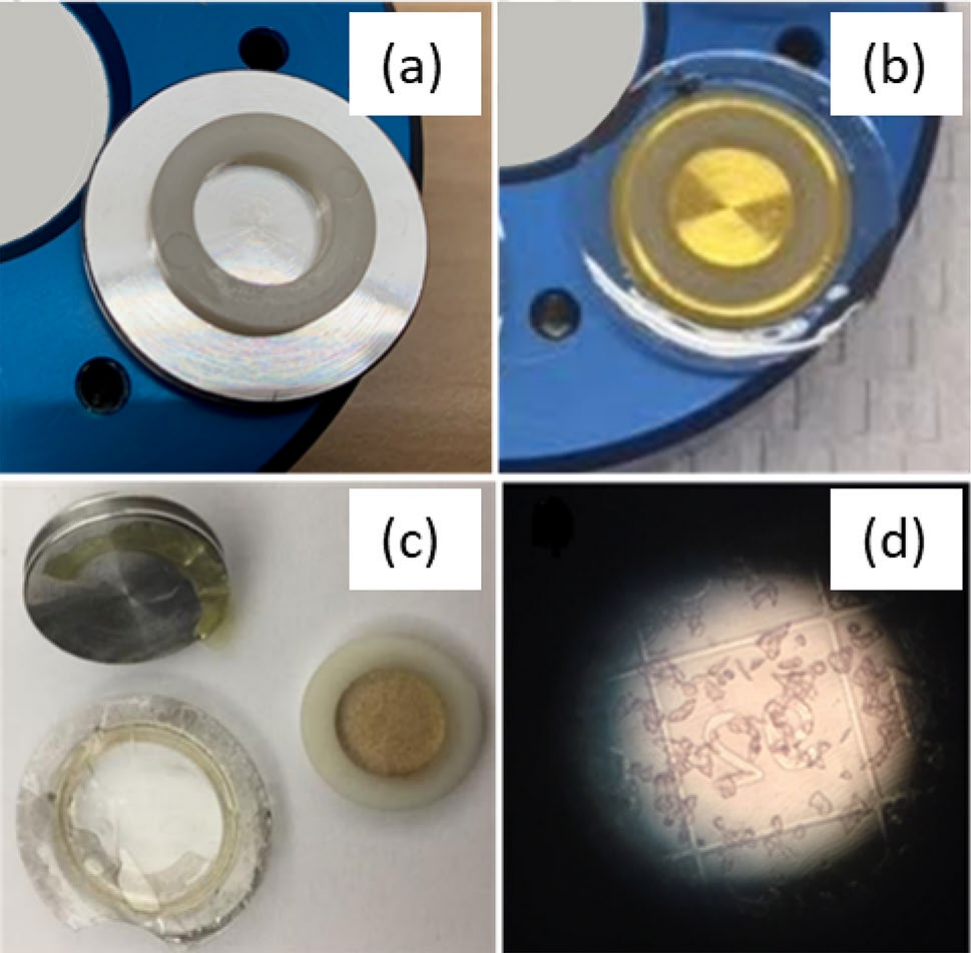
OSSM sample preparation. A thin ring plastic washer (**a**) is used as a sealing ring to form a container for the embedding medium. The coverslip was placed cell-side down onto the overfilled resin container (**b**). The coverslip was removed after resin polymerization by liquid nitrogen freeze fracture (**c**). Optical micrography of the top surface of the resin block showing imprinted coverslip markings and embedded cells (**d**).

After infiltration, samples that will undergo OSSM spin mill technique in the PFIB require a custom resin block geometry that is perfectly planar. To achieve this, we glue a very thin polyethylene washer directly onto the EM imaging stub to form a small open container (Fig. 11a). This container is then fully filled with resin as a mold for the polymerization. The coverslip with the imaged cells is then slowly placed cell-side-down onto the overfilled container such that the coverslip itself forms the final seal (Fig. 11b). After 24-h of thermal polymerization, a liquid nitrogen plunge is used to thermally shock and fracture the coverslip so that it may be removed from the resin (Fig. 11c). The top surface of the resin block can be checked under a light microscope after peeling the coverslip away. At this point, the cells are embedded into the top layer of the resin block, which is in the form of a flat disc for OSSM (or FIB/SEM, SBF/SEM, etc.) and imaging. The transferred pattern of the photoetched coverslip in the resin is used to identify the exact same cells (Fig. 11d) that were targeted in the iPALM imaging step.

### OSSM for serial PFIB/SEM imaging

Collection of OSSM data was performed on a prototype ThermoFisher Hydra PFIB DualBeam system operating with an oxygen plasma ion source (as opposed to the more conventional Xe^+^) which generates a mixture of molecular and atomic oxygen ions. The ion accelerating voltage or “landing energy” is selectable from 1–30 keV, but in these experiments a value of 12 keV was used as it was determined empirically to provide the highest SEM image quality on this sample. The resin block is positioned at the eucentric position of the system (Fig. 4a). Typically, samples are positioned in the FIB such that the sample surface is perpendicular to the angle of incidence of the ion beam. However, for OSSM the sample is tilted such that the FIB angle of incidence is 5–8 degrees from glancing relative to the sample surface (the plane of the cultured cell / former coverslip interface). The OSSM process consists of the following sequence: a brief oxygen FIB exposure of 10 s with beam current of 20 nA is performed over the desired area, with a milled diameter typically of approximately 400 µm (Fig. 4b). The stage is then rotated through a fixed angle, which is 30° in this experiment. This process is repeated 12 times until a full 360° rotation of the sample has been realized. Ion flux is delivered to the sample from several different azimuthal directions, which greatly reduces milling artifacts (such as “curtaining”) as compared to conventional top-down cross-sectional milling. One full rotation of milling constitutes a single “slice” in an analogy to FIB/SEM or SBF/SEM. The sample is then tilted back to the configuration for SEM imaging (sample surface normal to the SEM beam) to perform SEM imaging following each OSSM slice, very similar to the SBF/SEM acquisition step. This is automated and repeated many times to generate a sequence of images in z-space, which may be reconstructed into a 3D rendering of the same region that was imaged with the iPALM technique. Multiple ROIs can be defined within the larger FIB-milled area, and each of these sub-ROIs can be reconstructed as an independent 3D volume in the final 3D rendering. Figure 4c shows an example SEM image of the entire milled region following completion of the OSSM process. All the cells inside the ion-irradiated area (~ 1 mm-diameter circular region) are milled simultaneously during the FIB slicing. For this experiment, the imaging buffer lifetime imposed a time constraint on the iPALM step, and therefore only three different regions were imaged with iPALM before the sample was subjected to fixative. These iPALM-imaged areas are highlighted by arrows in Fig. 4c. It is noteworthy that this workflow enables analysis of numerous regions of interest (ROIs) simultaneously, and each ROI may be imaged with the SEM at much higher resolution as compared to the relatively low-resolution SEM images which are taken of the total milled area. ROIs being analyzed during OSSM can be added or discarded dynamically as the sample evolves eliminating time wasted on unimportant areas. Another distinguishing feature of OSSM is a decrease in mill time compared to normal FIB/SEM milling. Normal FIB/SEM CLEM with iPALM suffers from the need to perform FIB cross-sectioning and SEM imaging of each region individually, which is a time-consuming process that must be repeated serially for each ROI if there are multiple cells of interest. In the OSSM process, all the ROIs are FIB milled at the same time, although each ROI must be imaged with the SEM in series. Nevertheless, the ability to mill all ROIs simultaneously greatly improves the process throughput. The area of the sample which can be PFIB milled in the OSSM process is limited only by the field-of-view of the PFIB instrument (typically about 1 mm in size). This area is significantly larger than the areas which are usually milled in conventional slice-and-view FIB/SEM processing (typically a few 10 s of micrometers on each side). OSSM is also advantageous because it allows the experimentalist to capture all the detail that is found at the coverslip/cell interface in a very natural fashion as opposed to the normal cross-section, which requires the entire cell to be milled to reconstruct that full interface. It should be emphasized that OSSM captures the essential information about cell morphology, cell environment, and critically, the fiducial markers (Au nanorods) due to the preservation of the entire coverslip/cell interface.

### Data processing

As previously discussed, the gold nanorods that become embedded alongside the cell function as axial calibration for iPALM and directly guide the initial co-registration of the two data sets. Because the nanorods are represented in both EM and fluorescence imaging modalities, their positions are the most critical information to the correlation between the iPALM and OSSM datasets.

Because the total milled area is typically very large (hundreds of micrometers in diameter), conventional fiducial markers used for drift compensation and re-registration during SEM imaging are not useful. Therefore, images of individual ROIs are digitally stored and used as a reference images for the subsequent slice using pattern matching algorithms, which track the structural details from slice-to-slice. The result is a 3D SEM data set from multiple iPALM-imaged regions that are generated in an automated fashion and subsequently rendered and processed in the Amira visualization package.

The SEM data collected from PFIB were first processed with contrast match, image alignment, Gaussian filtering, and field of view crop functions from the stack file in Amira. In order to coarsely co-register the iPALM and SEM data sets, we first use gold fiducial markers, which are found in both iPALM and SEM images. The fiducial markers were typically evenly distributed around each cell and stabilized on the coverslip. After embedding, the gold nanorod fiducials exist in the first several SEM images along with the basal (i.e., close to the coverslip) portions of the cells. The disappearance of gold fiducials of known dimensions can assist in slice thickness estimates. Shown in Fig. 5 is an example of a 2D EM image that contains fiducial information and is aligned with a low-resolution, 2D iPALM projection image. The low-resolution iPALM image is a diffraction-limited rendering in which the nanorods appear as round disks of several hundred nm in diameter. In the SEM image, the markers are visible as black dots, each overlaid with the center of the corresponding disk in the iPALM image. After a reasonable manual coarse alignment was found via fiducial markers, the 3D data sets of both modalities were rigidly transformed in the X/Y 2D plane. A refinement of the registration was done by zooming into a location that covers tens of mitochondria.

Rendering both data sets as 3D volumes and performing minor manual registration adjustments of rotation based on cross-sectional images yielded the results in Fig. 9. The iPALM data here clearly showed the localization of TOMM20 protein bound to the outer membrane contour of mitochondria. We estimate the resolutions for our OSSM-SEM and iPALM based CLEM imaging to be ~ 20 nm and ~ 10 nm for the in-plane (x, y) and axial (z) dimensions, respectively. When processing iPALM localization data, we filtered the localizations to include those with localization precisions better than 0.1 pixel (~ 13.3 nm) in the x–y axis and ~ 7 nm in z (Fig. 8). PeakSelector^24^ reported that the remaining localizations, which represents the majority of the localizations, had mean single molecule localization precisions at 6.7 nm, 5.3 nm, and 3.2 nm for the x, y, and z axes, respectively. For SEM imaging, the voxel size was set as 6.5 nm × 6.5 nm × 5 nm. Considering the colocalization accuracy σ_c_ = sqrt (σ_SRM_^2^ + σ_EM_^2^), ideally σ_c_ is about 9.3 nm, 8.4 nm, 5.9 nm, for the x, y, and z axes. This corresponds to theoretical resolutions at 22 nm, 19 nm, and 14 nm for x, y, and z, consistent with visual inspections of the CLEM images (Figs. 8, 9). Of course, in reality, imaging resolution also depends on the sample labelling efficiency and labelling density in SRM, and imaging signal to noise ratio in EM.

## Conclusion

Although our ability to obtain the extensive 3D information from biological samples (cell, tissue…) by both LM and EM has grown extensively, methods for collecting high-content CLEM data are still in their infancy. Limitations are both due to each modality alone and due to drastic differences in the sample preparation and imaging steps. Here we present a novel 3D CLEM technique utilizing a reactive, focused oxygen beam to enable large area, high contrast serial block face imaging with reduced charging artifacts. The images collected from this so-called oxygen serial spin mill (OSSM) process can be correlated to 3D superresolution fluorescence images with high precision and structural authenticity. The workflow combines OSSM with interferometric photoactivation localization microscopy (iPALM) to create correlated superresolution fluorescence and SEM images in 3D. The technique was demonstrated by immunolabeling the outer mitochondrial membrane of human osteosarcoma cells and performing subsequent iPALM and OSSM imaging. The results indicate excellent correlation between the high resolution iPALM molecular images and the high contrast, ultrastructural SEM images produced by OSSM. Beyond imaging mitochondria, we anticipate the workflow demonstrated here to be broadly applicable to many more biological structures with no or slight modifications, for example by optimizing the fixation methods for the best comprise between structural preservations in EM or SRM.
